# New Perspectives on Criteria for the Determination of HCG Trigger Timing in GnRH Antagonist Cycles

**DOI:** 10.1097/MD.0000000000003691

**Published:** 2016-05-20

**Authors:** Xiaokun Hu, Yingyi Luo, Kejun Huang, Yubing Li, Yanwen Xu, Canquan Zhou, Qingyun Mai

**Affiliations:** From the Reproductive Medicine Center, The First Affiliated Hospital of Sun Yat-sen University, Guangdong, China.

## Abstract

The aim of this study was to investigate 2 quantification criteria to evaluate the developmental condition of follicles cohort and clarify their impacts upon the determining of human chorionic gonadotropin trigger timing and the reproductive outcome: the proportion of mature follicles in growing follicles cohort on the day of human chorionic gonadotropin trigger and the peak estradiol level per oocyte on the day of human chorionic gonadotropin administration.

Of the patients who underwent in vitro fertilization/ intracytoplasmic sperm injection-embryo transfer from 2011 to 2013, 492 controlled ovarian hyperstimulation cycles using gonadotropin-releasing hormone antagonists reaching the ovum pick-up and fresh embryo-transfer stage were included. Patients were divided into 3 groups according to their ≥17 mm/≥10 mm follicles ratio on the day of human chorionic gonadotropin administration (Low proportion: ≤30%, Middle proportion: 30%–60%, High proportion: ≥60%). Patients were divided into 5 groups according to their peak estradiol level/oocyte (Group A: <100 pg/mL per oocyte, Group B: 100–199 pg/mL per oocyte, Group C: 200–299 pg/mL per oocyte, Group D: 300–399 pg/mL per oocyte, Group E ≥400 pg/mL per oocyte) as well. Comparison among groups was made regarding ovarian stimulation characteristics, fertilization rate, good quality embryo rate, implantation, pregnancy, and live birth rates.

On the basis of ≥17 mm/≥10 mm follicles ratio, the number of oocyte retrieved in low proportion group is more than other 2 groups. Implantation rate, clinical pregnancy, and live birth rate in high proportion group were 25.8%, 42.7%, and 31.1%, respectively, which is highest in 3 groups, and statistical significance existed between high and middle proportion groups. When the division is based on peak estradiol level/oocyte, the number of oocyte retrieved of ≥400 pg/mL per oocyte Group was significantly lowest compared with the other 4 groups. Matured ovum rate, fertilization rate, and good quality embryos rate exhibited an increasing trend as the peak estradiol level/oocyte increased. While pregnancy rate, implantation rate, and live birth rate were found to be lower whenever estradiol/oocyte ratio exceeded 400 pg/mL per oocyte or less than 100 pg/mL per oocyte, and there is statistical difference.

Patients with the proportion of mature follicle reaching 60% on the day of human chorionic gonadotropin trigger and peak estradiol/oocyte level within 100∼399 pg/mL range can get a better pregnancy and implantation rate.

## INTRODUCTION

The timing of human chorionic gonadotropin (HCG) trigger for final maturation of targeted follicles is determined by many factors, such as follicle diameter, estradiol (E2) and progesterone level, the ratio of peak E2 to the number of oocytes larger than 14 mm, previous controlled ovarian hyperstimulation (COH) protocols, and embryo development. Timing of the onset of luteinization before oocyte retrieval is a terminal act that has an effect on oocyte quality and endometrium receptivity. Patients undergoing gonadotropin-releasing hormone (GnRH) antagonist-controlled cycles exhibit different profiles of follicular recruitment and serum sexual hormone secretion compared with GnRH agonist-controlled cycles.^1,2^ Although the criteria used for final oocyte maturation in GnRH antagonist stimulation cycle are as same as those in GnRH-agonist cycles.

The 2-stage pattern of follicular recruitment in antagonist-controlled cycles^2^ leads to a more unsynchronized developmental follicle profile than those obtained in agonist-controlled cycles. On the contrary, the GnRH-antagonist blocks pituitary GnRH receptor directly and inhibits follicle-stimulating hormone (FSH) and luteinizing hormone (LH) secretion without flare-up,^3^ which leads to serum E2 level decreasing or maintaining at a platform stage right after several days of GnRH antagonist administration. As a result, the serum estrogen levels are not consistent with the growth of follicles in GnRH antagonist protocol. Thus, such a heterogeneous cohort of follicles and different E2 elevation mode in GnRH antagonist-controlled cycles makes timing of HCG trigger much more complicated and important for the purpose of keeping the balance between maximizing the number of follicles cohort of a mature size and high quality of oocytes.

In most studies, HCG was administered when at least 3 follicles reach a diameter ≥17 mm^4–6^ on ultrasound. As a matter of fact, it is currently unclear of the ideal timing of HCG administration for GnRH antagonist in vitro fertilization/intracytoplasmic sperm injection (IVF/ICSI) cycles. As previously described by Ectors et al,^7^ a positive relationship between follicular size and the level of cytoplasmic maturation was observed. Postponing HCG administration and prolonging folliculogenesis to some extent yield relatively more target follicles containing competent oocytes with mature cytoplasm, and may exert positive effects on clinical outcomes.

In all the previous studies, the timing of HCG injection was based on the leading follicle size or simply postponed 1 or 2 days based on the leading follicles size and vague concept of consistent E2 levels regardless of the developmental condition of growing follicles cohort. In this retrospective study, we firstly utilized both the ≥17 mm/≥10 mm follicles ratio and the estradiol level per oocyte on the day of HCG (peak E2) to evaluate the follicles cohort developmental condition and determine the timing of HCG administration. These findings may help to clarify the impact of mature follicle proportion and average peak E2 level per oocyte upon the reproductive outcome. We attempted to provide more effective and objective criteria for determining the ideal HCG trigger timing and to obtain high-quality oocytes and higher pregnancy rate in GnRH antagonist-controlled cycles.

## METHODS

### Patients

Of the patients who underwent IVF/ICSI-ET at the Reproductive Medicine Center of The First Affiliated Hospital of Sun Yat-sen University from 2011 to 2013, 492 COH cycles with GnRH antagonist protocols reaching fresh embryo-transfer stage were included in the study. The exclusion criteria included endometrial polyps; premature progesterone elevation; preimplantation genetic diagnosis cycles; and nonfresh embryo transfer cycles. This study was approved by the Institutional Review Board of The First Affiliated Hospital of Sun Yat-sen University. All patients provided written, informed consent of assisted reproductive technology (ART) treatment.

### Stimulation Protocols

All patients underwent GnRH antagonist COH protocols in ART cycles. For the flexible multidose GnRH-antagonist protocol, gonadotropins (Gonal-F, Merck-Serono, Spain) were started on day 2 or 3 of the menstrual cycle. The initial dose of gonadotropin was individualized for each patient according to age, body mass index (BMI), antral follicle count, and/or previous responsiveness to ovarian stimulation. Further dose adjustments were performed on the basis of ovarian response, as evaluated using serum E2 measurement and follicular diameter by transvaginal ultrasound, obtained every 2 or 3 days. The GnRH-antagonist (cetrorelix, 0.25 mg daily, subcutaneously; Serono Laboratories, Geneva, Switzerland) was given an effective dose of 0.25 mg per day subcutaneously in a multiple dose protocol, from the day of mean diameter of dominant follicles larger than 10 mm onwards up to and including the day of HCG. Final oocyte maturation by HCG was triggered on the day when at least 3 mature follicles ≥17 mm were observed by ultrasonography. Transvaginal ultrasound guided oocytes retrieval was carried out 36 hours after HCG trigger. Routine IVF/ICSI was then performed as appropriate. The luteal phase was supported with either 40 to 60 mg progesterone intramuscularly (Gestone; Ferring, USA) daily or 600 mg vaginal micronized progesterone soft gel capsules (Utrogestan; Besins Iscovesco, Petah Tikva, Israel) in 3 separate doses daily. Progesterone prolonged releasing vaginal gel (Crinone; Merck Serono, Switzerland) 90 mg daily was supplemented as luteal support as well.

Good-quality embryos were defined as equal-sized, symmetric blastomeres with <10% fragmentation under inspection in a microscope. Fertilization rate was defined as the percentage of fertilized embryos (2PN) in all the mature oocytes. The implantation rate was the proportion of embryos transferred resulting in an intrauterine gestational sac. The embryos were transferred after 3 days of ovum pick-up and up to 3 embryos were transfer. Pregnancies were confirmed 3 or 4 weeks after ET, when the serum HCG was elevated. A clinical pregnancy is defined by the presence of 1 or more gestation sacs in the uterus.^8^

### Clinical Data Collection

A blood test was performed on the 2nd to 5th day of the menstrual cycle before the treatment to determine FSH, LH, and E2 activity. During treatment, patients’ information was documented, including age, duration of gonadotropin administration, total gonadotropin dose, estradiol and progesterone levels on the HCG day, number of oocyte retrieved, and numbers of good quality embryos and transferred embryos. Matured oocyte rate, fertilization rate, implantation rate, clinical pregnancy rate, and live birth rate were calculated.

### Statistical Analysis

The statistical analyses were performed using SPSS 13 package program (SPSS Inc, Chicago, IL, USA). Categorical data were expressed as number and percentage. Continuous variables were given as mean ± standard deviation if they presented normal distribution. Statistics was given as median and range when the assumption of normality is violated. One-factor analysis of variance (one-way ANOVA) was applied for the comparison between groups for continuous data. Homogeneity of variances was tested and Student–Newman–Keuls (SNK) test was used with equal variances. When the variances were unequal, Tamhanes T2 test was applied as indicated. Pearson Chi-square analysis (χ^2^ test) was used as well to compare the data among different groups. A *P* value of less than 0.05 was considered statistically significant with Bonferroni corrections.

## RESULTS

A total of 492 consecutive IVF/ICSI cycles were evaluated. Clinical characteristics of the ART cycles in the antagonist group are presented in Table 1. Patients were divided into 3 groups according to their ≥17 mm/≥10 mm follicles ratio on the day of HCG administration (Low proportion: ≥17 mm/≥10 mm ratio ≤30%, Middle proportion: ≥17 mm/≥10 mm ratio 30%–60%, High proportion: ≥17 mm/≥10 mm ratio ≥60%). No difference existed within the 3 groups with respect to age, basal FSH and LH level, and total gonadotropin dosage in antagonist cycles.

**TABLE 1:**
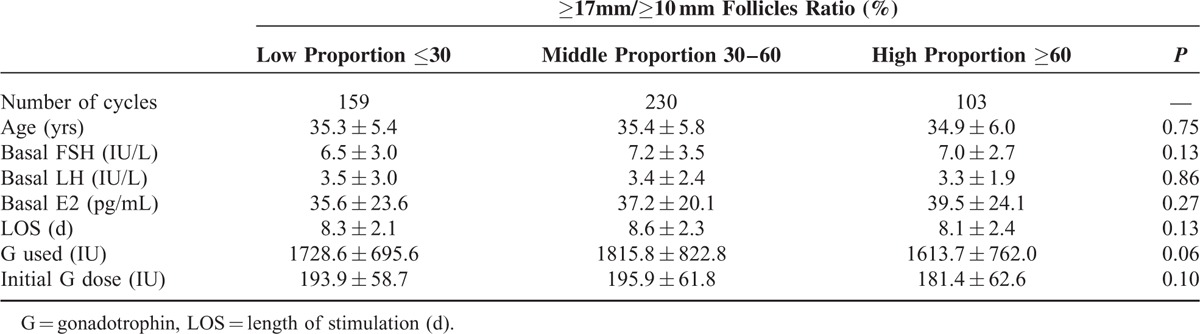
Clinical Characteristics of GnRH Antagonist ART Cycles

**FIGURE 1:**
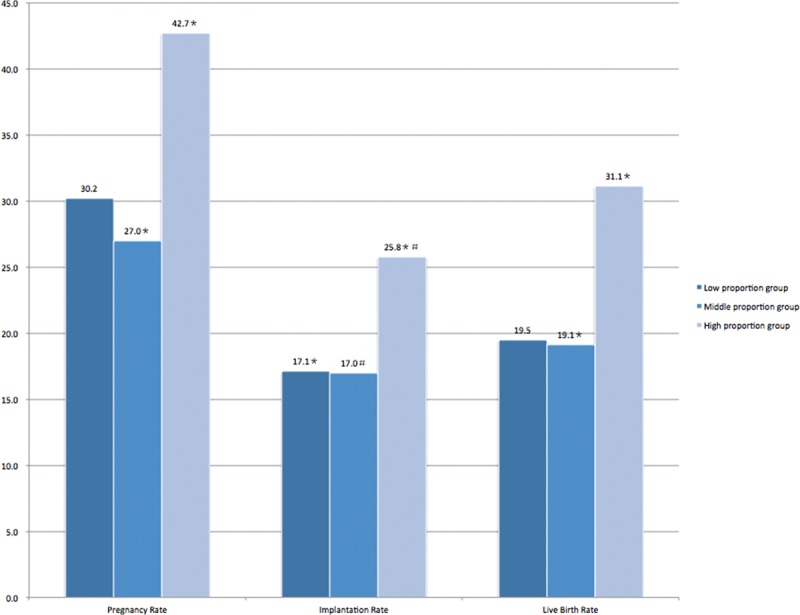
Pregnancy outcomes of GnRH antagonist cycles in different ≥17 mm/≥10 mm follicles ratio groups^, ∗,#^*P* < 0.05. Values with the same superscript with respect to pregnancy rate, implantation rate, and live birth rate, respectively, are significantly different. The clinical pregnancy rate Middle proportion group: 27.0% vs. High proportion group: 42.7%, *P* = 0.004; The implantation rate Low proportion group: 17.1% vs. High proportion group: 25.8%, *P* = 0.011; Middle proportion group: 17.0% vs. High proportion group: 25.8%, *P* = 0.007; The live birth rate Middle proportion group: 19.1% vs. high proportion group: 31.1%, *P* = 0.016.

Ovarian response characteristics and reproductive outcomes of the ART cycles in GnRH antagonist group are presented in Table 2 and Figure 1. In Table 2, the number of oocyte retrieved in the low proportion group (9.2 ± 6.3) was significantly higher than those in the middle (7.6 ± 6.0) and high (7.2 ± 6.6) proportion groups. Peak E2 level per oocyte retrieved was highest in high proportion group with a large proportion of large follicles. The peak E2 level per oocyte retrieved in the low proportion group (242.5 ± 121.8 pg/mL) was significantly lower than those in the middle (285.4 ± 165.4 pg/mL) and high (312.1 ± 194.5 pg/mL) proportion groups. The matured ovum rate in the middle and high proportion group was higher than that in the low proportion group (Low proportion group: 85.1%, Middle proportion group: 89.2%, High proportion group: 87.9%), and statistical significance existed between the low and middle proportion group. Fertilization rate and good quality embryo rate was comparable among the 3 groups. The number of fresh embryos transferred in the low proportion group (2.3 ± 0.7) was meaningfully higher than those in the middle (2.2 ± 0.7) and high (2.0 ± 0.7) proportion groups. The implantation rate of ≥17 mm/≥10 mm groups was 25.8% (54/209) in the high proportion group, which was significantly higher than the low proportion group (17.1%, 64/375) and middle proportion group (17.0%, 84/495). The clinical pregnancy rate of ≥17 mm/≥10 mm groups was 30.2% (48/159) in the low proportion group, 27.0% (62/230) in the middle proportion group, and 42.7% (44/103) in the high proportion group, and statistical significance existed between middle and high proportion group. Similarly, the live birth rate exhibited an increasing trend as the ≥17 mm/≥10 mm follicles ratio increases and that of the high proportion group (31.1%, 32/103) was significantly higher than the middle proportion group (19.1%, 44/230).

**TABLE 2:**
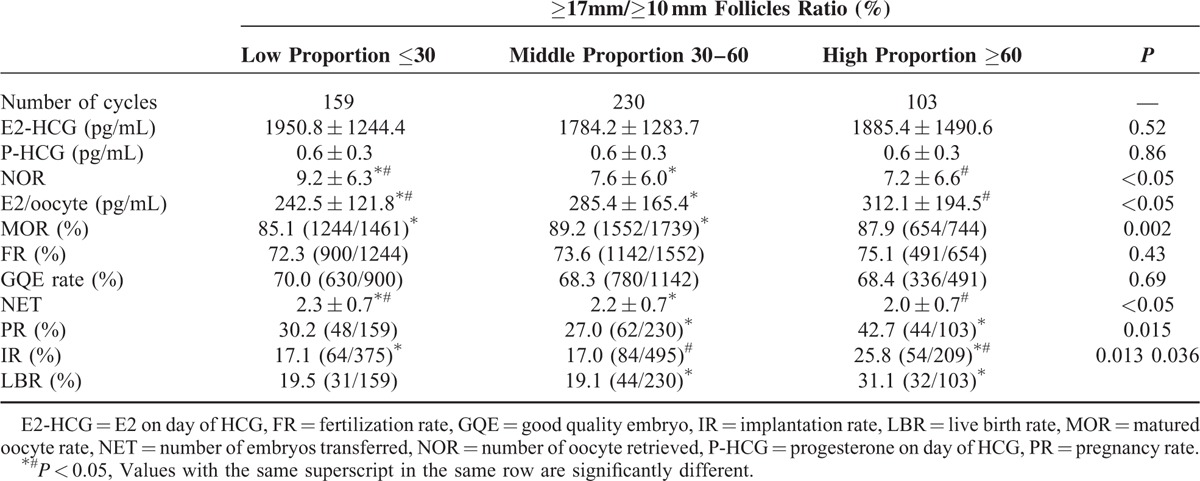
Ovarian Response Characteristics and Reproductive Outcomes of GnRH Antagonist ART Cycles

**FIGURE 2:**
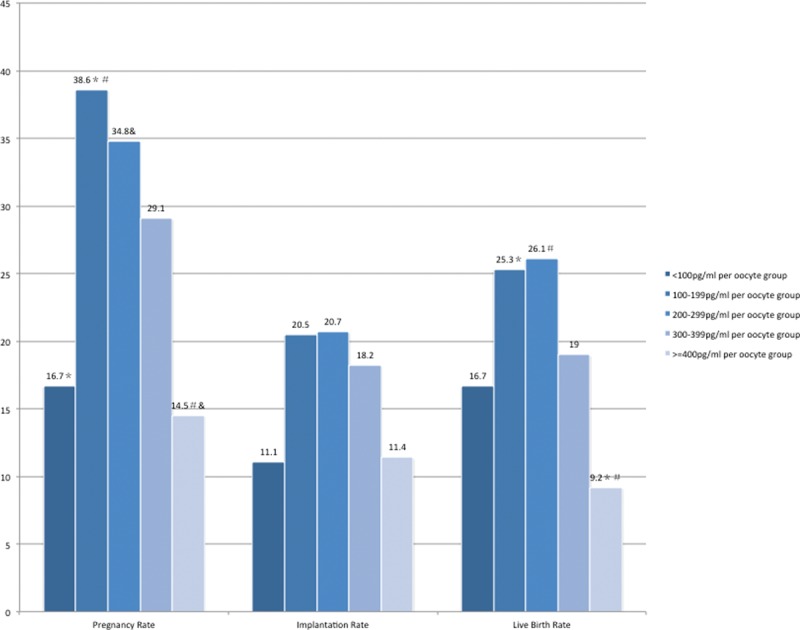
Pregnancy outcomes of GnRH antagonist cycles in different peak E2 level/oocyte groups, ^∗, #, &^*P* < 0.05. Values with the same superscript with respect to pregnancy rate, implantation rate, and live birth rate, respectively, are significantly different. The clinical pregnancy rate 100 to 199 pg/mL per oocyte Group: 38.6% vs. ≥400 pg/ml per oocyte Group: 14.5%, *P* = 0.000; 200 to 299 pg/mL per oocyte Group: 34.8% vs. ≥400 pg/mL per oocyte Group: 14.5%, *P* = 0.001; <100 pg/mL per oocyte Group: 16.7% vs. 100 to 199 pg/mL per oocyte Group: 38.6%, *P* = 0.002; The live birth rate 100 to 199 pg/mL per oocyte Group: 25.3% vs. ≥400 pg/mL per oocyte Group: 9.2%, *P* = 0.004; 200 to 299 pg/mL per oocyte Group: 26.1% vs. ≥400 pg/mL per oocyte Group: 9.2%, *P* = 0.003.

Patients were further divided into 5 groups according to their peak E2 level/oocyte (Group A: <100 pg/mL per oocyte, Group B: 100–199 pg/mL per oocyte, Group C: 200–299 pg/mL per oocyte, Group D: 300–399 pg/mL per oocyte, Group E ≥400 pg/mL per oocyte). Ovarian response characteristics and reproductive outcomes with different peak E2 level/oocyte group are summarized in Table 3 and Figure 2. No difference existed within the 5 groups with respect to age, basal FSH and LH level, and total gonadotropin dosage.

**TABLE 3:**
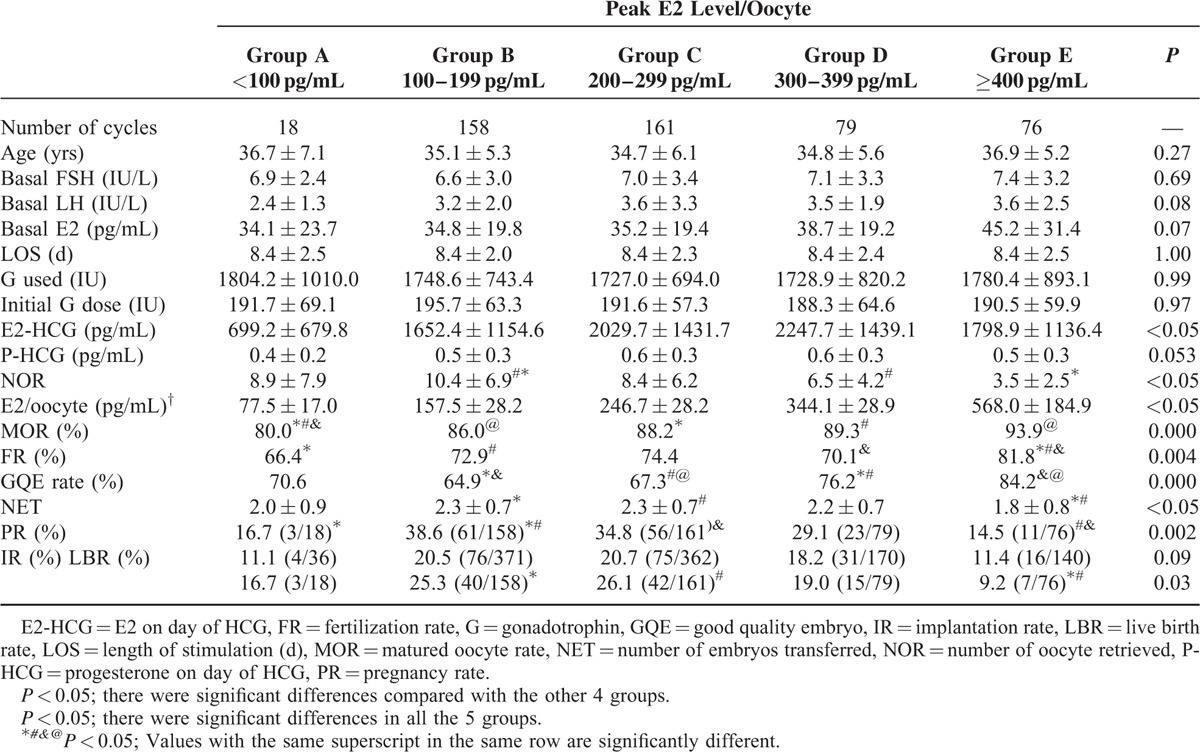
Ovarian Response Characteristics and Reproductive Outcomes With Different Peak E2 Level/Oocyte in GnRH Antagonist Group

The number of oocyte retrieved of 300 to 399 pg/mL per oocyte Group (6.5 ± 4.2) and ≥400 pg/mL per oocyte Group (3.5 ± 2.5) were significantly lower than that of 100 to 199 pg/mL per oocyte Group (10.4 ± 6.9). Matured ovum rate increases as the peak E2 level/oocyte increases, and that of <100 pg/mL per oocyte Group (80.0%, 128/160) was meaningfully lower than those in 200 to 299 pg/mL per oocyte Group (88.2%, 1198/1358, *P* = 0.003) and 300 to 399 pg/mL per oocyte Group (89.3%, 461/516, *P* = 0.002). What's more, the matured ovum rate of ≥400 pg/mL per oocyte Group (93.9%, 247/263) was significantly higher than those in <100 pg/mL per oocyte Group (80.0%, 128/160, *P* = 0.000) and 100 to 199 pg/mL per oocyte Group (86.0%, 1416/1647, *P* = 0.000).

The fertilization rate displayed an increasing tendency as the peak E2 level/oocyte increases. The fertilization rate of ≥400 pg/mL per oocyte Group (81.8%, 202/247) was significantly higher than those in <100 pg/mL per oocyte Group (66.4%, 85/128, *P* = 0.001), 100 to 199 pg/ml per oocyte Group (72.9%, 1032/1416, *P* = 0.003), and 300 to 399 pg/mL per oocyte Group (70.1%, 323/461, *P* = 0.001).

The good quality embryos rate of 300 to 399 pg/mL per oocyte Group (76.2%) and ≥400 pg/mL per oocyte Group (84.2%) were significantly higher than 100 to 199 pg/mL per oocyte Group (64.9%) and 200 to 299 pg/mL per oocyte (67.3%). However, the number of fresh embryos transferred of 100 to 199 pg/mL per oocyte Group (2.3 ± 0.7) and 200 to 299 pg/mL per oocyte (2.3 ± 0.7) were significantly more than that of ≥400 pg/mL per oocyte Group (1.8 ± 0.8). In addition, the implantation, pregnancy, and live birth rate were higher in 100 to 199 pg/mL per oocyte group, 200 to 299 pg/mL per oocyte group, and 300 to 399 pg/mL per oocyte group than <100 pg/mL per oocyte Group and ≥400 pg/mL per oocyte Group (Group A: 11.1%/16.7%/16.7%, Group B: 20.5%/38.6%/25.3%, Group C: 20.7%/34.8%/26.1%, Group D: 18.2%/29.1%/19.0%, Group E: 11.4%/14.5%/9.2%). Pregnancy rate of 100 to 199 pg/mL per oocyte Group and 200 to 299 pg/mL per oocyte Group were significantly higher than that of ≥400 pg/mL per oocyte Group as shown in Figure 2. Significant difference also existed between <100 pg/mL per oocyte Group and 100 to 199 pg/mL per oocyte Group. The live birth rate of ≥400 pg/mL per oocyte Group was significantly lower than that of 100to 199 pg/mL per oocyte Group and 200 to 299 pg/mL per oocyte Group.

## DISCUSSION

This is the first study to use quantification criteria to evaluate the mature status of follicles cohort and choose the ideal timing of HCG trigger in GnRH antagonist protocol. The mature follicles proportion and elevated E2 level per oocyte were used to evaluate the developmental condition of numerous follicles in various sizes. In this retrospective study, comparison of implantation and pregnancy rate among different proportion of mature follicles group and different level of peak E2/oocyte was made to find out an ideal timing of HCG trigger. The higher proportion of mature follicle within 100∼399 pg/mL E2 level per oocyte on the HCG day was associated with a higher pregnancy and implantation rate in GnRH antagonist protocol.

There is no solid basis on current established HCG trigger timing criteria and it is not a strict criterion. Even some researchers believed that the determining of HCG trigger timing should rely on the experience of clinicians. It is controversial about the effect of postponing HCG trigger timing in GnRH antagonist cycles. In a randomized controlled trial (RCT) of antagonist cycles, Kolibianakis et al^9^ delayed HCG administration by 2 days after the time that 3 follicles had reached 17 mm in diameter and observed an increase in early pregnancy losses and decrease in ongoing pregnancy rates in the delayed group. Another RCT research in 2012, focused on the timing of HCG administration in IVF protocols using GnRH antagonists,^10^ demonstrates that delaying HCG administration had no significant negative impact upon morphological quality of embryos, availability of surplus embryos for freezing or pregnancy outcomes. Besides, postponing HCG may enable increased flexibility of cycle scheduling to avoid weekend procedures.

With the deepening of the understanding of cohort follicles development, the purpose of COH is providing high-quality oocytes with nuclear and cytoplasmic maturation. While how to evaluate oocytes’ maturation status in developing follicles cohort? As we know, follicle diameter and E2 level are 2 useful clinical indexes used to judge whether the oocyte in growing follicles is mature or not. Thus, we selected 2 objective criteria including mature follicle proportion and average peak E2 level per oocyte to analyze their effects on IVF outcomes in patients undergoing GnRH-antagonist COH protocols and try to provide more effective and objective criteria for determining the ideal HCG trigger timing than the theoretically proposed fixed criterion of “at least 3 follicles are more than 17 mm of diameter.”

Several studies had reported that postponing HCG administration for inducing final follicular maturation by 1 to 2 days did not influence reproductive outcome. The main point we need to focus on is the whole developmental condition of growing follicles cohort other than to consider whenever the HCGs were injected on the day on which at least 3 follicles reached 17 mm or 2 days later.

Kolibianakis et al^9^ reported that in GnRH antagonist cycles, the proportion of follicles with diameters 11 to 15 mm, 15 to 17 mm, and17 mm or more were 35%, 15%, and 30%, respectively, on the day of HCG administration. These follicles of different developmental stage produced different competent oocytes. It has been well established in several domestic and model species that the proportion of competent oocytes increases with follicular size. Many studies have demonstrated that follicular size is positively related to the ability of oocyte to be fertilized and to develop, and those oocytes from large follicles tend to develop high-quality embryos.^7,11,12^ The difference in pregnancy and implantation rate among 3 groups indicates that embryos originating from different size group follicles might have different long-term developmental competence.

In contrast to the result of present studies, the number of oocyte retrieved in the low proportion group was significantly higher than the other 2 groups, while the mature oocyte rate is lowest in the low proportion group. The fertilization rate and good quality embryo rate was comparable among the 3 groups. The oocytes in some oversize follicles might either degenerate or decrease in quality, which made the oocyte retrieval decreasing. On the contrary, a premature harvest yields oocytes that are not yet cytoplasmically mature even though they readily undergo nuclear maturation and fertilization, which has a detrimental effect on embryo development.^13^

In agreement with the result of present study, as the ≥17 mm/≥10 mm follicles ratio increases, peak serum E2 level and peak E2 level per oocyte retrieved were increased. The increase of E2 level is not always consistent with the enlarging follicle diameter in GnRH antagonist cycle. Fleming ^2^ demonstrated that the rate of increase of estradiol concentration was reduced at GnRH antagonist administration, and in some cases, the concentration may even show a short-term decrease. Akman et al^14^ observed that serum E2 levels were significantly lower in the antagonist group on cycle day 2, HCG day, and oocyte retrieval day than the agonist groups. Lindheim and Morales^15^ found a decline in serum estradiol in almost one-third of the cycles after GnRH antagonist administration as well. All the previous studies indicated that we need another range of E2 level to evaluate the maturation of follicles in GnRH antagonist cycle. In the latter stage of COH, multiple follicles with various sizes are present and contributing to a different degree to E2 production. Moreover, the dynamics of LH secretion are different between the GnRH agonist and antagonist cycle.^2^ It makes the concept of E2 level per follicle in GnRH antagonist stimulated cycles complex and vague. Orvieto et al^16^ suggested that patients undergoing GnRH antagonist protocol should reach a peak E2 level/oocyte within the 100 to 200 pg/mL range in order to gain a higher pregnancy rate.

In our retrospective study, the number of oocyte retrieved was significantly lowest when average peak E2 level/oocyte was higher than 400 pg/mL. The lost oocyte in ≥400 pg/mL per ooccyte group may be partially explained by the concomitant high intrafollicular level of androgen, which has a deleterious effect on follicular maturation and development, thus enhance ovarian granulose cell apoptosis^17^ or, alternatively, by a high proportion of degenerative oocytes.^18,19^

Matured ovum rate, fertilization rate, and good quality embryos rate showed an increasing tendency as the peak E2 level/oocyte increases. Pregnancy rate, implantation, and live birth rate were found to be lower whenever peak E2 level/oocyte exceeded 400 pg/mL or less than 100 pg/mL. The consistent E2 levels are believed to indicate follicular health. Accordingly, Yang et al^20^ found that IVF cycles using GnRH-agonist protocol with an elevated peak E2 level/oocyte exceeding 350 pg/mL/oocyte correlated with lower pregnancy and implantation rates. In our study, it should be noted that although the pregnancy and implantation rate were lowest in ≥400 pg/mL per oocyte group, the mature ovum rate, fertilization rate, and high-quality embryos rate were highest. The low implantation and pregnancy rate may be due to the effect of supraphysiological serum estradiol concentrations and GnRH antagonist administration on endometrial receptivity.^21,22^ So that, when the peak E2 level per oocyte exceed 400 pg/mL, patients should avoid fresh embryo transfer and all embryos should be cryopreserved for frozen-thawed embryos transfer. The peak E2 level/oocyte less than 100 pg/mL indicates that the oocytes are immature. The low pregnancy and implantation rate might be due to the less competent oocytes with immature cytoplasm. Even though they readily undergo nuclear maturation, it has a detrimental effect on fertilization and embryo development.^13^

Our findings showed that when the proportion of >17 mm follicles is more than 60%, the patients can get a higher pregnancy and implantation rate in GnRH antagonist protocol. At the same time, patients should keep the peak E2 level/oocyte within a range of 100∼399 pg/mL in order to gain better IVF outcome, lower pregnancy, and implantation rates and could be expected if the range is exceeded. While for the patient with E2 level/oocyte higher than 400 pg/mL, all embryos should be considered cryopreserved in case of low endometrial receptivity and low pregnancy rate.

The limitations of this study include its retrospective nature and the small number of patients in the different groups. In the future, it may be useful to plan prospective randomized studies with an equal number of patients and to assess the proper timing of HCG administration during COH in in vitro fertilization cycles using GnRH antagonist protocols.

## CONCLUSION

As far as we know, this is the first study to investigate both the proportion of mature follicles and the ratio of peak E2 to the number of oocytes retrieved to clarify the proper timing of HCG administration in GnRH-antagonist COH protocols. Patients with the proportion of mature follicle reaching 60% on the day of HCG trigger and peak estradiol/oocyte level within 100∼399 pg/mL range can get a better pregnancy and implantation rate in GnRH antagonist protocol. Lower pregnancy rate could be expected whenever E2/oocyte ratio exceeds 400 pg/mL per oocyte in fresh embryo transfer GnRH antagonist cycles. We provide new and objective criteria to determine more appropriate timing of HCG trigger for high-quality oocytes and higher pregnancy rate in GnRH-antagonist COH protocols. It is also the first time to clarify relationship between the E2 level per oocyte and mature follicles proportion, which offers new clue to evaluate cytoplasmic maturation status of oocytes and helps clinicians determine optimal HCG trigger timing in GnRH-antagonist COH protocols.
